# The importance of proper bioinformatics analysis and clinical interpretation of tumor genomic profiling: a case study of undifferentiated sarcoma and a constitutional pathogenic *BRCA2* mutation and an *MLH1* variant of uncertain significance

**DOI:** 10.1007/s10689-015-9790-3

**Published:** 2015-02-25

**Authors:** Elizabeth Varga, Elizabeth C. Chao, Nicholas D. Yeager

**Affiliations:** 1 Division of Hematology/Oncology/Bone Marrow Transplant, Nationwide Children’s Hospital, 700 Children’s Drive, Columbus, OH USA; 2 Department of Pediatrics, The Ohio State University College of Medicine, Columbus, OH USA; 3 Ambry Genetics and Division of Genetics and Genomics, Aliso Viejo, CA USA; 4 Department of Pediatrics, University of California, Irvine, CA USA

**Keywords:** Tumor profiling, Lynch syndrome, Sarcoma, Hereditary breast and ovarian cancer syndrome, Incidental findings

## Abstract

Next-generation sequencing (NGS) technology is increasingly utilized to identify therapeutic targets for patients with malignancy. This technology also has the capability to reveal the presence of constitutional genetic alterations, which may have significant implications for patients and their family members. Here we present the case of a 23 year old Caucasian patient with recurrent undifferentiated sarcoma who had NGS-based tumor analysis using an assay which simultaneously analyzed the entire coding sequence of 236 cancer-related genes (3769 exons) plus 47 introns from 19 genes often rearranged or altered in cancer. Pathogenic alterations were reported in tumor as the predicted protein alterations, *BRCA2* “R645fs*15″ and *MLH1* “E694*”. Because constitutional *BRCA2* and *MLH1* gene mutations are associated with Hereditary Breast Ovarian Cancer Syndrome (HBOCS) and Lynch syndrome respectively, sequence analysis of DNA isolated from peripheral blood was performed. The presence of the alterations, *BRCA2* c.1929delG and *MLH1* c.2080G>T, corresponding to the previously reported predicted protein alterations, were confirmed by Sanger sequencing in the constitutional DNA. An additional DNA finding was reported in this analysis, *MLH1* c.2081A>C at the neighboring nucleotide. Further evaluation of the family revealed that all alterations were paternally inherited and the two *MLH1* substitutions were in *cis*, more appropriately referred to as *MLH1* c.2080_2081delGAinsTC, which is classified as a variant of uncertain significance. This case illustrates important considerations related to appropriate interpretation of NGS tumor results and follow-up of patients with potentially deleterious constitutional alterations.

## Introduction

Increasingly, oncologists and pathologists are using next-generation sequencing assays to identify potential therapeutic targets that may help successfully treat malignancy. Multiple oncology trials to assess the utility of next-generation sequencing (NGS) assays are underway [1]. Although NGS assays are intended to provide information about acquired genomic alterations in tumor DNA, it is acknowledged that constitutional alterations may also be identified. Here we present the case of a 23 year old Caucasian patient with recurrent undifferentiated sarcoma, where tumor profiling reported pathogenic alterations in *BRCA2* and *MLH1*. Constitutional analysis confirmed a pathogenic *BRCA2* alteration and led to reclassification of the *MLH1* alteration as a variant of uncertain significance.

## Materials and methods

A 20 year old Caucasian female first presented with a progressively enlarging, painless left sided anterior chest wall mass above her left breast. Computed tomography was performed demonstrating a 3 × 3 cm mass arising anterior to her left second rib with erosion into the rib and extension into the pleural space. Surgical removal of the tumor was performed with pathology demonstrating a high grade undifferentiated sarcoma. The patient received post-operative radiotherapy (6000 cGy) and adjuvant chemotherapy with ifosfamide and doxorubicin.

Eleven months after completion of the initial treatment course, the patient re-presented with severe lower back and left leg pain and a new palpable skull mass. Imaging confirmed new masses, including a 5 cm tumor in her left sacral ala and a 4.5 cm lesion in her left parietal skull. The sacral lesion was re-biopsied and confirmed recurrence of her original undifferentiated sarcoma. Chemotherapy was reinitiated with three cycles of ifosfamide, carboplatin, and etoposide (ICE) along with palliative radiotherapy (3900 cGy). Because she was judged to be at high risk for subsequent recurrence, she received maintenance chemotherapy. She completed 9 months of treatment before a pleural based lesion was noted in her right thorax. Surgical removal was performed to remove the lesion, and pathology was consistent with her prior resections.

In an effort to identify therapies that might directly target the molecular profile of her tumor, a commercial next-generation sequencing assay as described by Frampton et al. [2], was ordered. This assay simultaneously analyzes the entire coding sequence of 236 cancer-related genes plus 47 introns from 19 genes often rearranged or altered in cancer. All classes of genomic alterations (base substitutions, insertions and deletions, copy number variations and rearrangements) are detectable with this assay.

## Results

Two pathogenic genomic alterations were reported from tumor testing, listed on the report as *BRCA2* “R645Efs*15″ and *MLH1* “E694*”. The report stated that “the *BRCA2* mutation is expected to lead to premature truncation of the Brca2 protein prior to the area of Rad51 binding and the DNA binding domain. This mutation is therefore predicted to be inactivating…Therefore, in the appropriate clinical context, testing for the presence of germline mutations in BRCA2 is recommended.” The report stated that the *MLH1* mutation, “E694*”, “observed in this tumor results in a truncation of the 756-amino acid Mlh1 protein at amino acid 700. This mutation is expected to result in the loss of part of the C-terminal domain required for Pms2 binding and formation of the MutLalpha complex. Truncation of MLH1 at amino acid 749–750 impairs the ability of Mlh1 to act in error correction, checkpoint signaling and Pms2 interaction and stabilization…Germline MLH1 mutations are associated with Lynch syndrome, which is manifested by increased risk of a number of cancers, especially colorectal carcinoma. Therefore, in the appropriate clinical context, germline testing of MLH1 is recommended.”

There are no clinically available therapies to target these gene mutations. Poly (ADP-ribose) Polymerase (PARP) inhibitors, which facilitate DNA double stranded break repair, are currently being studied in clinical trials and recent studies suggest that cells with inactivation of Brca2 may be sensitive to PARP inhibitors [3].

**Fig. 1:**
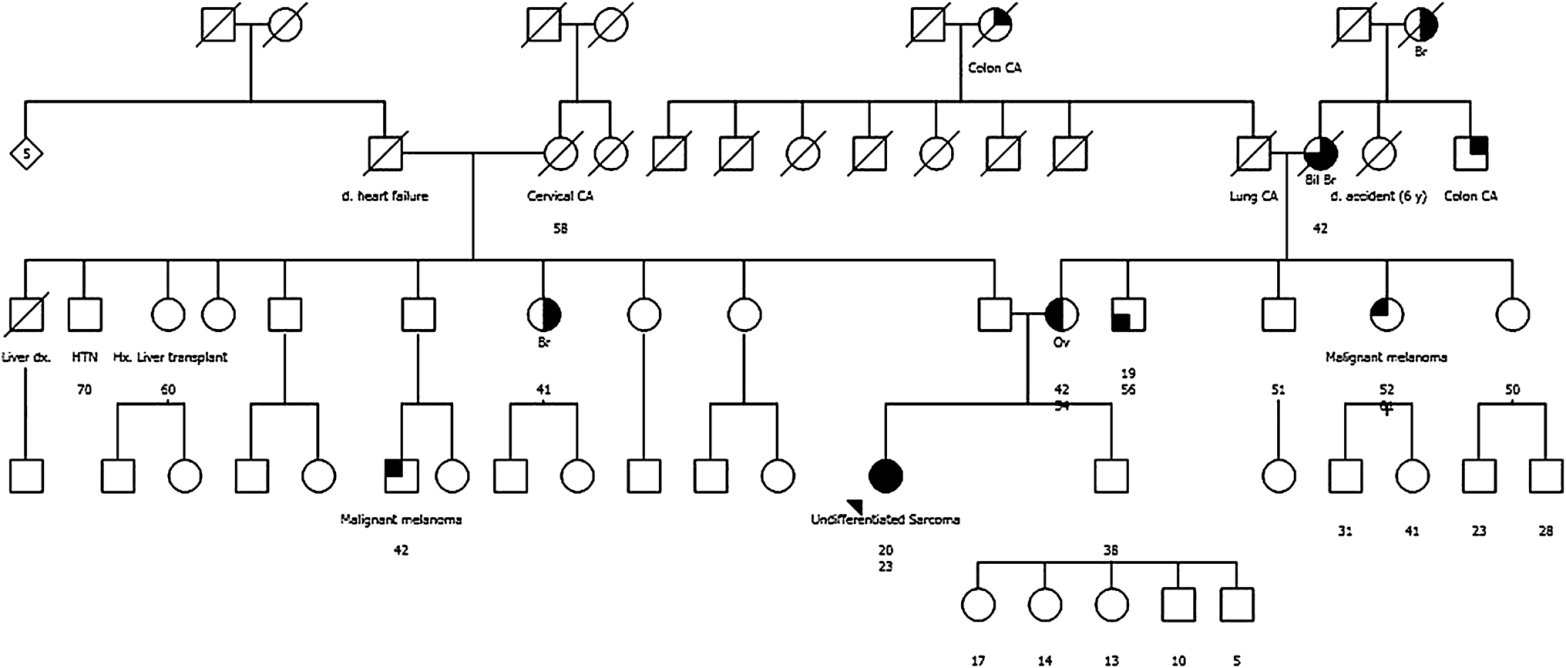
Pedigree. Age at diagnosis listed above current age. *Ov* ovarian, *Bil Br* bilateral breast, *Br* breast

Because the presence of constitutional mutations in *BRCA2* and/or *MLH1* has significant implications for the patient and family members, a genetic consultation was recommended. A four generation pedigree was obtained by a licensed genetic counselor (Fig. 1). The patient’s mother reported ovarian cancer at age 42, discovered incidentally when hysterectomy was performed for endometriosis. A maternal uncle had testicular cancer at age 19, a maternal aunt had recurrent basal cell carcinoma (total six; onset 50 s), and the maternal grandmother had bilateral breast cancer resulting in double mastectomy at age 42. Colon, breast, lung and prostate cancer at typical ages of onset (60–70 s) was reported in maternal great grandparents and great aunts and uncles. The patient’s paternal family history included one aunt with unilateral breast cancer at age 41, a paternal first cousin with melanoma (diagnosed 42) and unspecified abdominal tumor, and a paternal grandmother with cervical or uterine cancer at age 58. The ethnicity of the family was reported as German and Irish (maternal) and unspecified European (paternal) with no consanguinity. No one in the family was known to have had genetic testing, or immunohistochemical/microsatellite instability testing of tumors previously. Pathology records for family members were requested but were not able to be obtained.

Given the mother’s diagnosis of ovarian cancer at age 42 and the maternal grandmother’s diagnosis of bilateral breast cancer, the maternal family history meets National Comprehensive Cancer Network (NCCN) clinical criteria for HBOCS [4, 5]. The family history does not meet Amsterdam I, II or revised Bethesda criteria for Lynch syndrome. The paternal family history does not meet criteria for HBOCS or Lynch syndrome.

After genetic counseling, the patient elected to have DNA analysis performed on a blood sample for targeted analysis of the *BRCA2* and *MLH1* alterations. The tumor profiling report was sent along with the patient’s blood sample to a CLIA-certified laboratory specializing in constitutional genomic analysis. Through consultation with the tumor testing laboratory, it was determined the protein alterations reported through tumor profiling were predicted based on the following DNA findings: c.1929delG in *BRCA2* and c.2080G>T in *MLH1*. Testing was performed using targeted PCR-based amplification of the relevant coding exon followed by dideoxy termination (Sanger) sequencing. This testing confirmed the presence of these alterations in DNA isolated from peripheral blood, suggesting that the abnormalities were constitutional in nature. In addition, a variant in the neighboring basepair of *MLH1* (c.2081A>C) was detected which in and of itself would be predicted to result in the amino acid substitution p.Glu694Ser. Retrospective review of the tumor profiling report found this alteration included in the appendix as *MLH1* “E694S” under “variants of unclear significance (VUS)” along with seven other alterations. That this alteration was neighboring to the reported c.2080G>T, or the phase (*cis* or *trans*), was not specifically noted on the tumor profiling report.

Constitutional genetic testing for the patient’s *BRCA2* and *MLH1* alterations was recommended for all first-degree relatives. The patient’s mother was tested through a CLIA-certified laboratory and found to be negative for the *BRCA2* mutation, and both *MLH1* alterations. Given the family history, repeat analysis was requested and produced the same results. Subsequently, the patient’s father was tested, revealing that he had the *BRCA2* c.1929delG pathogenic mutation, as well as both *MLH1* alterations. Confirmation that both *MLH1* changes were paternally inherited and this located on the same chromosome (*cis*) led to revised nomenclature and re-interpretation. Because Sanger sequencing was used in the setting of confirmatory testing, phase was determined through familial analysis. However, retrospective analysis of NGS-based sequencing data also confirmed the presence of these alterations in *cis* (data not shown).

The two previously reported neighboring single basepair subsitutitons in *MLH1* actually represent an insertion-deletion (indel) event which is most accurately described as c.2080_2081delGAinsTC. This alteration is in-frame and is predicted to result in the substitution of a serine for glutamic at position 694, but not to lead to a premature protein truncation. These amino acids have similar properties. This variant has not been reported in the literature, locus-specific databases, nor population based cohorts in the Database of Single Nucleotide Polymorphisms (dbSNP), NHLBI Exome Sequencing Project (ESP) or the 1000 Genomes Project. To date, this alteration has been detected with an allele frequency of approximately 0.01 % in the testing laboratory’s clinical cohort (greater than 13,000 alleles tested including the proband and her father). Using the MAPP-MMR variant prediction tool, this alteration received a score of 3.010; scores between 3.0 and 5.0 are considered borderline/inconclusive [6]. Based on the current evidence, this alteration present in both the proband and her father is best classified as a variant of uncertain significance.

The patient’s mother went on to have comprehensive genetic testing with a NGS panel of 23 genes (*ATM, BARD1, BRCA1, BRCA2, BRIP1, CDH1, CHEK2, EPCAM, MLH1, MRE11A, MSH2, MSH6, MUTYH, NBN, NF1, PALB2, PMS2, PTEN, RAD50, RAD51C, RAD51D, STK11,* and *TP53)*. Full gene sequencing and analysis of all coding domains plus at least 5 bases into the 5′ and 3′ ends of all the introns and untranslated regions (5′UTR and 3′UTR) was performed for 22 of the 23 genes (excluding *EPCAM*). Gross deletion/duplication analysis was performed for all 23 genes. This panel was negative for pathogenic alterations.

## Discussion

There is increasing recognition that “tumor-only” NGS may reveal constitutional mutations, creating ethical quandaries [7, 8]. In this case, tumor sequencing led to the discovery of a pathogenic constitutional *BRCA2* mutation, and a *MLH1* alteration, initially misclassified as pathogenic, but later reinterpreted as a variant of uncertain significance. This highlights the importance of evaluating a DNA sequence variant not in isolation in the context of surrounding sequence, as well as the value of using NGS-based data to determine phase when multiple nearby variants are identified. The reclassification of the *MLH1* alteration created specific genetic counseling challenges.

Initially, the patient was counseled that she had pathogenic constitutional alterations of *BRCA2* and *MLH1*, based on tumor and constitutional sequencing results. Several case reports describe the phenotype of patients with digenic mutations [9–12] and a review of these cases suggests there is a significantly increased risk for cancer, including a high risk of multiple primary tumors [12]. Since surveillance guidelines are not established for those with digenic mutations, National Comprehensive Cancer Network (NCCN) Screening Guidelines for HBOCS and Lynch syndromes was initially advised for our patient [4, 5]. Reclassification of the *MLH1* alteration led to revised genetic counseling regarding the patient’s cancer risks and risks to relatives and altered management recommendations (HBOCS guidelines only).

Another unanticipated finding was that despite the patient’s maternal family history meeting clinical criteria for HBOCS, testing revealed that the *BRCA2* mutation was paternally inherited. The maternal family history remains unexplained. The father was informed of his associated risks of breast, prostate and other cancers [13, 14]. Genetic testing is relevant for his at risk relatives, including his sister who was diagnosed at 41 with breast cancer, since studies suggest that woman with *BRCA2* mutations have an increased risk of contralateral breast cancer in the absence of intervention [15] and that the risk of ovarian, primary peritoneal and fallopian tube cancer is 11–18 % by age 70 [16, 17]. However, the father remains reluctant to communicate results to relatives, creating conflict regarding duty to warn at risk relatives, and patient autonomy and confidentiality.

In addition to illustrating complex genetic counseling issues that may arise in tumor only gene sequencing, this case highlights important technical considerations. Laboratories performing tumor sequencing may apply different protocols, bioinformatics approaches and pathogenicity classifications when analyzing data [18]. Nomenclature used in somatic mutation reporting may not be consistent nationally accepted guidelines set out by the Human Genome Variation Society, the American College of Medical Genetics and Genomics (ACMG), the College of American Pathologists, or others [19, 20]. Disparate analysis and reporting practices may lead to confusion when constitutional testing is performed.

In this case, targeted Sanger sequencing was performed to confirm the *BRCA2* and *MLH1* mutations, both reported initially as pathogenic. A known limitation of Sanger sequencing is that this methodology is unable to differentiate phase of two reported alterations, even when they are located in the same amplicon or sequence read. Phase has routinely been determined by follow-up testing of family members, most notably parents or offspring. In our case, parental testing provided additional information to properly classify the *MLH1* alteration as a variant of uncertain significance.

It is valid to ask why the *MLH1* was misclassified initially on the tumor profiling report. The phase of the two alterations should be identified using NGS, as long as both alterations are located within the same sequencing read (typically within 50–200 bp depending on the protocol). When the laboratory was contacted, they discovered that their bioinformatics pipeline had indeed appropriately flagged the alteration, prompting pathology review. However, human error led to misreporting in this case (personal communication). The tumor profiling laboratory amended the original report, after a visual review of NGS data.

As NGS sequencing is increasingly performed within clinical and research settings, unanticipated findings will continue to increase. As discussed by other authors, guidelines need to be developed to provide recommendations regarding efficient pre-test counseling and informed consent prior to tumor-only testing, as well as post-test disclosure, while carefully considering the original intent of testing [7, 8, 18]. As illustrated in this case, it will be important to consider the possibility of potential follow-up of more than one alteration that may be present constitutionally. Recommendations will also need to consider conflicts that may arise between patient autonomy and confidentiality and duty to warn family members of known genetic risks.
